# An optimized method for tissue glycogen quantification

**DOI:** 10.14814/phy2.15195

**Published:** 2022-02-18

**Authors:** Kyle J. Schaubroeck, Brooks P. Leitner, Rachel J. Perry

**Affiliations:** ^1^ Departments of Cellular & Molecular Physiology and Internal Medicine (Endocrinology) Yale School of Medicine New Haven Connecticut USA

## Abstract

Mobilization of glycogen, the short‐term storage form of glucose, is the body's first defense against hypoglycemia and is critical for energy provision during high intensity exercise. Therefore, to advance metabolic research, it is critical to be able to accurately measure glycogen concentrations, including during a prolonged fast and other glycogen‐modulating interventions. Unfortunately, prior enzymatic methods of glycogen detection have been plagued by poor detection in the lower range, high sample mass requirements, and complicated and/or expensive protocols which introduce substantial technical variability into the measured glycogen concentrations. To address these limitations, here we report a streamlined and versatile glycogen extraction protocol coupled with an optimized phenol‐sulfuric acid quantification protocol. With this method, we demonstrate how glycogen can be extracted from only 20 mg of tissue with one centrifugation step and quantified with a highly precise (Intra‐assay %CV ranges from 5–10%) and sensitive (proportionality constant for glycogen = 0.07279 A.U./µg) assay. The cost of all materials equates to ~10 cents per sample. Therefore, this method represents an attractive means of assessing *ex vivo* tissue glycogen content including at the extremes of glycogen concentrations.

## Graphical abstract

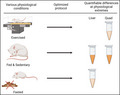

## 1 INTRODUCTION

The role of glycogen in maintaining blood glucose concentrations within the relatively narrow range that is characteristic of normal physiology has long been appreciated. Glycogen was first described by Claude Bernard more than 150 years ago (Young, 1957) and represents a crucial player in the maintenance of glucose homeostasis in health and disease (Adeva‐Andany et al., 2016; Berg et al., 2021). Easily mobilized to glucose, glycogen breakdown is the body's first defense against hypoglycemia and earliest means of supplying energy under conditions of metabolic stress. Therefore, when studying metabolic physiology and pathophysiology, it is of great importance to be able to measure glycogen content reliably and in a cost‐effective manner.

Three of the most used glycogen quantification methods are the amyloglucosidase method, the anthrone method, and the phenol‐sulfuric acid method. Amyloglucosidase hydrolyzes glycogen to glucose, and the glucose from glycogen can be measured as the difference between free glucose (in sample that is not hydrolyzed) and glucose in sample treated with amyloglucosidase (Huijing, 1970; Yi et al., 2016). While the method works well under glycogen replete conditions when large sample volumes are available, reliance on measurement of glucose concentrations in two samples compounds experimental uncertainty and is unreliable at very low glycogen concentrations or when free glucose concentrations are high. In addition, enzyme costs are relatively high, and duration of functional activity can be short with frequent freeze‐thaw cycles. The anthrone method utilizes alkaline digestion of glycogen, precipitation of undigested proteins with excess acid, and addition of the anthrone reagent to detect glucose spectrophotometrically (Roe & Dailey, 1966). This method is subject to the same limitations regarding free glucose as the amyloglucosidase. Considering free glucose in skeletal muscle and liver does not change in the same direction as glycogen content under various conditions, the degree of variability attributed to free glucose can contribute a large source of variability.

In the phenol‐sulfuric acid method, sulfuric acid is used to dehydrate glycogen to 5‐hydroxymethylfurfural, which then reacts with phenol to generate an orange‐colored solution, the absorbance of which can be measured spectrophotometrically. Side reactions have previously been observed using this method. Rao et al. observed that sulfonation of phenol reduced color intensity, and solved the issue by adding phenol after furfural formation completed (Rao & Pattabiraman, 1989). Rao et al.’s solution suggests potential side reactions can be controlled by carefully selecting conditions.

A variety of phenol‐sulfuric acid protocols have been reported in the literature (Lo et al., 1970; Masuko et al., 2005; Michel et al., 1956; Rao & Pattabiraman, 1989; Rasouli et al., 2014; Taylor, 1995) (Table 1).

**TABLE 1:** Comparison between our method and prior iterations of the phenol‐sulfuric acid method

	Drawbacks	Our Method
Rao and Pattabiraman *Anal*. *Biochem*. (Rao & Pattabiraman, 1989) DuBois et al. *Anal*. *Chem*. (Michel et al., 1956) Lo et al. *J*. *Appl*. *Physiol*. (Lo et al., 1970) Taylor *Appl*. *Biochem*. *Biotechnol*. (Taylor, 1995)	Utilizes milliliters of sample, leaving no material for replicates or other uses	Utilizes microliters of sample. Allows for technical replicates or leaves sample for other uses
Masuko et al. *Anal*. *Biochem*. (Masuko et al., 2005)	Requires two water baths – one for cooling and one heating (near boiling – 90°C) Requires users to place and retrieve a microplate in a water bath without injury or contaminating samples	No water baths, reducing equipment required Easier and safer to use ‐ No temperature manipulations
Rasouli et al. (2014)	4 pipetting steps per sample instead of the traditional 3 steps	3 pipetting steps per sample ~ Twice the sensitivity Comparable precision

In general, most agree that adding phenol after sulfuric acid provides the best results; however, a recent protocol added phenol then sulfuric acid and still managed to obtain reproducible results (Masuko et al., 2005; Rasouli et al., 2014; Taylor, 1995). The literature also suggest that careful control of the reaction temperature is essential for precision and sensitivity, however, many protocols handle the initial temperature differently. Most previous work on optimizing signal for the phenol‐sulfuric acid method has utilized a “one variable at a time” approach where all variables are held constant and only the variable of interest is changed. Such approaches are inefficient and can lead to an incomplete understanding of how all variables interact. Therefore, we utilized a multifactor approach where we changed multiple variables simultaneously in a controlled way. With this method, we generated a mathematical model that can predict the behavior of the method and utilized it to find conditions with good precision and some of the highest sensitivity for the method. Here we describe an optimized protocol for glycogen quantification using the phenol‐sulfuric acid method. In addition, the cost of all materials equates to ~10 cents per sample. We anticipate that this method will be of utility particularly when examining glycogen content in muscle and other tissues in which glycogen concentrations are low at baseline.

## 2 MATERIALS AND METHODS

### 2.1 Chemicals

95 – 98% sulfuric acid (Sigma 258105)

Referred to as “concentrated sulfuric acid.”

5% (w/v) phenol solution – made from phenol crystals (Sigma 242322) and DI water. Aliquoted and stored at −20°C. Thawed and stored at 5°C.

Powdered Glycogen from bovine liver (Sigma G0885) – solutions made with DI water.

200 proof (100%) ethanol (Fisher Scientific A40920)

Sodium sulfate (Na_2_SO_4_) crystals (Sigma S9627) made into a 9.5% (w/v) solution using DI water.

Sodium hydroxide crystals (NaOH) (Sigma 655104) made into a 0.5 M solution with DI water.

1 M HCl solution (Sigma H9892)

Abcam Glycogen Assay Kit II (colorimetric) (Abcam ab169558)

**FIGURE 1:**
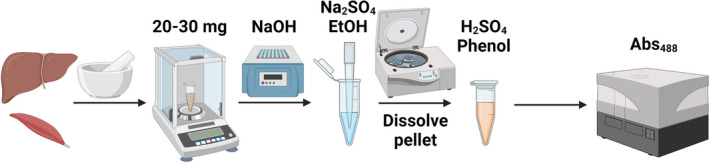
Method workflow. This figure, created with BioRender.com, shows the main steps in the glycogen analysis method proposed here

A summary of the study workflow is shown in Figure 1.

### 2.2 Animals

All animal protocols were approved by the Institutional Animal Care and Use Committee at Yale University. Biological samples were obtained from (1) 48 h fasted mice which had undergone an infusion of [U‐^13^C_6_] glucose at a low infusion rate (1.0 mg/kg/min for 120 min) that does not perturb systemic metabolism (Perry et al., 2018), (2) 48 h fasted rats which were not infused with tracer, (3) glucose loaded mice which had been gavaged with 100 ul of 50% dextrose solution 45 min prior to sacrifice, and (4) mice immediately following a bout of high‐intensity interval training (HIIT) on the treadmill. The one‐hour HIIT protocol consisted of alternating treadmill speeds of 12 m/min. and 17 m/min. every two minutes. HIIT was performed three days after two days of treadmill acclimation (20 min) and an incremental maximal speed test. The maximal speed of the exercised mice was 23.6 ± 2.7 m/min.

### 2.3 Optimizing heating of reagents

Two methods of heating phenol were tested. The “heat of dilution” method was conducted by mixing 90 µl of DI water with 39 µl of 5% (w/v) phenol in a 1.5 ml Eppendorf tube. Then, 300 µl of concentrated sulfuric acid was added rapidly directly to the solution by reverse pipetting. The reaction sat for 20 min before 250 µl of sampled was transferred to a microplate and the absorbance read at 490 nm. The “heating” method was conducted by adding 90 µl of DI water to an Eppendorf and rapidly adding 300 µl of concentrated sulfuric acid. The solution was allowed to sit for 15–20 min before being transfer to a water bath set to 80°C. After 1 min, the tube was removed and 39 µl of 5% (w/v) phenol was added. The solution sat for 10 min before 250 µl of sample was transferred to a microplate and the absorbance read at 490 nm. Glycogen samples were assayed in a similar way, using 90 µl of a 10 mg/dl glycogen stock in place of DI H_2_O and 39 µl of DI H_2_O instead of phenol. The temperature was set at 90°C. Controls containing only water and concentrated sulfuric acid were measured to establish a baseline. All protocols were run in at least duplicate.

### 2.4 Optimizing the order of addition

90 µl of DI water was added to a 1.5 ml Eppendorf tube. Then, 300 µl of concentrated sulfuric acid was added followed by 39 µl of 5% (w/v) phenol. The reverse order – 39 µl of 5% (w/v) phenol followed by 300 µl of concentrated sulfuric acid – was also tested. All reactions were allowed to proceed for 30 min before 250 µl of sample was transferred to a microplate and the absorbance read at 490 nm. Once the optimized protocol was selected, a spectrum containing glycogen, phenol, and concentrated sulfuric acid was collected. All protocols were run in triplicate.

### 2.5 Generation of the multifactor response surface model

A standard central composite design for three independent variables was generated. We selected our independent variables to be the volume of 5% (w/v) phenol solution (V_phenol_), volume of concentrated sulfuric acid (V_H2SO4_), and volume DI water (V_H2O_). All reactions contained 50 µl of 10 mg/dl glycogen solution. With this setup, the total volume will vary. This variation is acceptable for two reasons. First, the total mass of glycogen, and therefore number of moles of glycogen, was held constant in the reaction. Second, any effects volume has on signal will be captured in the proportionality constant.

To generate the central composite design, we selected a high and low volume of each independent variable and encoded them (+1 and −1, respectively). Encoded variables use the same nomenclature as the corresponding independent variable but include an asterisk.

The encoded values were used to create an embedded factorial design. Factorial designs require testing the independent variables at all possible combinations of high and low values. In our setup that means 2^3^ different reactions. The factorial design was augmented with axial points. Axial points set one independent variable to either +2^(3/4)^ or −2^(3/4)^ while the other independent variables are set to 0. All possible axial points were tested. The center point – defined as all coded independent variables set to 0 – was run six times. In total, 20 chemical reactions were conducted. Each reaction was allowed to proceed for 30 min after initiation. None of our reactions had volumes were less than 300 µl, so we transferred 250 µl of sample to a microplate and the absorbance read at 490 nm.

All but two center point reactions were randomly ordered by a random number generator. The two center point reactions were selected to begin and end the sequence. This design choice means that center points start, finish, and are dispersed throughout the experiment, allowing the process to be monitored for instability occurring during the experiment.

The data were analyzed in GraphPad Prism (version 9.2) and initially fit to a full quadratic model with all possible interaction terms. For each parameter, Prism tests the hypothesis that the true population value of that parameter is zero. Terms with a *p* < 0.05 were selected for removal. We verified the need to remove each term by comparing the initial quadratic model to the selected model using the difference in the Akaike information criterion (AIC).

### 2.6 Experimental verification of the model's accuracy

We tested our model's accuracy at three different conditions (Table 2). First, we maximized our model to determine a condition expected to contain high signal. This condition is far from experimental data, so the accuracy of the prediction is expected to be low. The second condition was selected because it was an initial estimate of conditions expected to provide good precision. The third condition was randomly selected.

**TABLE 2:** Conditions selected to test accuracy

Condition	V_phenol_*	V_H2SO4_*	V_H2O_*
1	−1.7	−1.1	−1.682
2	0.7	0	−1.682
3	−0.7	0.5	−1.082

All conditions were run by adding 50 µl of a 10 mg/dl glycogen solution followed by the appropriate volume of water. Concentrated sulfuric acid was then added rapidly via reverse pipetting and afterward phenol was added. All conditions were run four times. The difference between the two theoretical means and the experimental mean was analyzed along with the 95% confidence interval.

### 2.7 Selection of sensitive and precise conditions

We have derived a function for the absorbance: OD_490 nm_ = R(A, B, C). Calculus gives us the total differential of the function: dR = (∂R/∂A)·A + (∂R/∂B)·B + (∂R/∂C)·C.

We can approximate this as the error. The ‘worst‐case’ scenario would be when all the terms add and none of the error cancels. For this, we added the magnitudes: |ΔR| ≈ |(∂R/∂A)·A| + |(∂R/∂B)·B| + |(∂R/∂C)·C|.

These can be turned into relative error to consider the amount of signal produced: |ΔR/R| ≈ |1/R|·(|(∂R/∂A)·A| + |(∂R/∂B)·B| + |(∂R/∂C)·C|).

We approximated a 1% error in the uncoded values. For example, assume the volume of phenol was expected to be 50 µl. A 1% error would yield a value of 49.5 µl. Once these parameters are coded, the difference is 0.05. All calculations for finding sensitive and precise conditions used the difference in encoded values.

We utilized Excel's Solver Add‐In to find a numerical solution with the following criteria:

The total volume had to be at least 300 µl.

The volume of water added was constrained to 0 µl.

We settled on 45 µl phenol, 300 µl of concentrated sulfuric acid, and 0 µl of water.

We tested the precision and sensitivity of the condition by generating a standard curve using both glycogen and glucose. We used a large range of standards, ranging from 0 to 25 µg. The standard curve only included points under an absorbance of 1.6 A.U. to prevent deviation from Beer's law.

Outliers were identified using Prism's ROUT method with Q = 1%. Two points on the glycogen curve – one at 10 µg and one at 20 µg – were identified but only the 20 µg point was removed. The absorbance of the removed point matches those of an adjacent standard. During one experimental run, one reaction did not receive glycogen when the other reactions received it and this mistake was corrected later in the protocol. We believe this reaction received the incorrect amount of glycogen during this correction. With statistical reason, an experimental deviation, and a mechanism for an error, the point was removed. There was no experimental reason or mechanism why the second point could be an outlier, so the data was included in the main analysis. An analysis of the data with the outlier is included.

Precision was quantified by determining the intra‐assay coefficient of variation (%CV) which is defined as the standard deviation divided by the mean. The %CV is only valid for variables where “0” means 0. In this case, that means 0 absorbance means 0 material. Therefore, all points were blanked by subtracting the y‐intercept from the corresponding standard curve.

### 2.8 Optimized precipitation steps

A control mixture of glucose and glycogen was created at a concentration of 60 mg/dl glucose and 40 mg/dl glycogen. This mixture was subjected to the same extraction protocol as all other samples. We mixed 50 µl of the control mixture with 200 µl of 0.5 M NaOH and heated in a boiling water bath (100°C) for 30 min with frequent agitation to simulate a homogenization step. We wrapped the lids in foil to prevent evaporation and to keep the lids from popping open.

After the ‘homogenization step’ the tubes were allowed to cool to room temperature for about 10 min. Then, 50 µl of 9.5% (w/v) Na_2_SO_4_ was added to each sample to act as a co‐precipitant. Next, we precipitated the glycogen by adding 2 volumes (600 µl) of absolute (100%) ethanol. The tubes were centrifuged at 2000 g for 10 min to pellet the glycogen and remove low molecular weight carbohydrates. Samples underwent a variable number of rounds of centrifugation to determine the number of rounds of centrifugation required to remove free glucose. The supernatant was discarded, and the pellet formed during centrifugation is the first precipitation. The pellet was redissolved by vortexing in 100 µL of DI H_2_O and acidified with 5 µl of 1.0 M HCl. We added 2 volumes (200 µl) of absolute ethanol, vortexed, and centrifuged at 2000 g for 10 min. The supernatant was discarded. This pellet is the 2nd precipitation. We repeated the redissolving, precipitation, vortexing, and centrifugation to generate precipitations 3 and 4.

All precipitated glycogen / Na_2_SO_4_ pellets were washed with 50 µl of absolute ethanol. The tubes were then turned upside down to dry for 5 to 10 min. Next, the pellets were dissolved in 250 µL of DI water and then assayed for total carbohydrate content using the optimized phenol‐sulfuric acid assay (50 µl sample, 300 µl concentrated sulfuric acid, and 45 µl of 5% (w/v) phenol) with glucose as the standard curve.

Each precipitation number was tested 4 times. One control mixture stock solution was used for the duration of the experiment to reduce errors arising from sample preparation. Therefore, all variability observed comes from setting up and running the procedure.

### 2.9 Checking for sodium sulfate interference

Either 50 µl of water or 50 µl of 9.5% (w/v) Na_2_SO_4_ was added to 1.5 ml Eppendorf tubes. Each tube received 300 µl concentrated sulfuric acid and 45 µl of 5% (w/v) phenol. The solution sat for 30 min before we transferred 280 µl to a microplate to read the absorbance at 488 nm.

### 2.10 Verification of the procedure by extraction from tissues

We sampled liver and quadriceps muscle from *ad lib* fed, healthy rats. Each tissue was ground to a fine powder under liquid nitrogen with a mortar and pestle. Each tissue was sampled 3 times, 20–30 mg each sample. All tubes used were pre‐chilled with dry ice and each tissue sample was stored on dry ice until use. Samples containing 50 µl of the same glucose/glycogen control mixture from the optimized precipitation steps were also prepared.

All samples underwent the optimized precipitation procedure up to the 1st precipitation: 200 µl of NaOH, 30 min boiling (100°C), 50 µl of 9.5% (w/v) Na_2_SO_4_, 600 µl of ethanol, centrifugation at 2000 g for 10 min, and 5–10 min drying. We omitted the final ethanol wash step to simplify the procedure.

The pellet from the control mixtures was dissolved in 250 µl of DI H_2_O. We dissolved the quad samples in 500 µl of DI H_2_O, and the liver samples in 1000 µl of DI H_2_O. Heating the tubes likely caused changes in shape as the lids were prone to leaking during vortexing. Therefore, we covered the lids of the tubes with parafilm when vortexing the pellet back into solution.

Glucose and glycogen in each sample were quantified using the Abcam Glycogen Assay Kit II (colorimetric) according to the manufacturer's directions. In addition to the purified control mixture, the unpurified control mix was also quantified.

The total amount of carbohydrate was also analyzed using the optimized phenol‐sulfuric acid assay. We suspected our samples were too concentrated for the assay. However, since we didn't know the concentration, we did not want to dilute the entire sample and risk over dilution. We therefore added sample to a 1.5 ml Eppendorf. We used 50 µl of control solution, 25 µl of quadriceps solution, and 5 µl of liver solution. All volumes were then brought to 50 µl using DI water. These samples received 300 µl concentrated sulfuric acid, and 45 µl of 5% (w/v) phenol and waited for 30 min before 280 µl was read at 488 nm. A standard curve using glycogen was prepared. Each sample was run in duplicate and averaged to get the estimate for that sample. The data were analyzed according to the equation:

$$\frac{m_{\text{glycogen}}}{m_{\text{tissue}}}=\frac{\left(m_{\text{curve}}/V_{\text{assayed}}\right)*V_{\text{sample}}}{m_{\text{tissue}}}$$

where m_curve_ is the mass determined from the standard curve, V_assayed_ is the volume of sample used in the phenol‐sulfuric acid assay, V_sample_ is the volume of DI water used to dissolve the purified glycogen pellet, and m_tissue_ is the mass of tissue used to make the sample.

## 3 RESULTS

### 3.1 Selecting optimized reaction conditions

**FIGURE 2:**
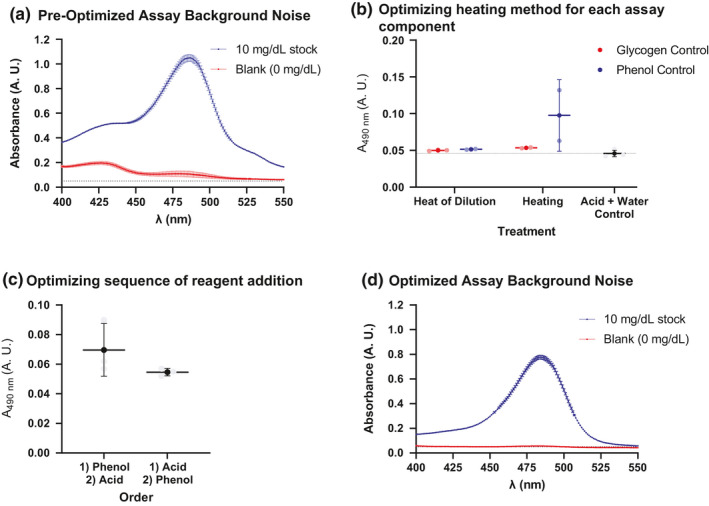
Method optimization data. (A) The pre‐optimized protocol was done by mixing carbohydrate, phenol, and then sulfuric acid, followed by heating. The background curve (red) is much higher than the background produced in the absence of assay mixture (black dashed line) A ‘shoulder’ peak appears around 425 nm. (B) To test how different components in the reaction respond to heat, we prepared several samples with either glycogen/acid or phenol/acid. These reactions contained only 2 out of the 3 components needed for a complete reaction. That way, we could observe the components in their semi‐natural environment but without a complete reaction obscuring any unwanted reactions. The acid/water control contained acid and water instead of phenol or glycogen. This defined the background without any reactions. (C) Once we optimized the heating step, we also tested the order to add reagents. (B) and (C) show the mean ± standard deviation. (D) The post optimized procedure mixes carbohydrate, sulfuric acid, and then phenol all at room temperature. The background of the blank solution (red) matches the expected background in the absence of sample. The shoulder peak at 425 nm has also disappeared. All points were run in triplicate except the heated phenol sample in (B) which was run in duplicate

In our early work with the phenol‐sulfuric acid method, we observed blanks – solutions without glycogen – would have significant and variable absorbance. Sometimes, solutions would turn pale‐orange and have an OD of about 0.1 A.U. when measured at 490 nm. When observing the spectra of glucose samples, we noticed a ‘shoulder’ peak appearing in the lower 400 nm range (Figure 2A).

We suspect the variable absorbance and shoulder peak could be due to background reactions, as phenols are known to decompose into orange color products. Additionally, the primary intermediate in the phenol‐sulfuric acid reaction, 5‐hydroxymethylfurfural (HMF), is known to decompose into products that can absorb in the low 400 nm range (Shao et al., 2019).

We first tested the heating of the reagents. We tested two options: heating from an external heat bath (“heating”) and heating from the addition of acid (“heat of dilution”). We found the heat of dilution gave more reproducible results and values closer to the concentrated sulfuric acid +water control (Figure 2B).

We also tested the order to add the phenol. We tested two options: the protocol from Rasouli et al. (2014) (add phenol then acid) and the protocol from Masuko et al. (2005) (add acid then immediately add phenol). Both methods gave low background signal but the Masuko et al. protocol gave more reproducible values (Figure 2C). Addition of acid causes a violent release of heat, occasionally causing the solution to briefly boil or for vapor to be released. We postulate the violent heat release could cause the phenol, if present, to form small quantities of unwanted products or to boil out of the solution. We selected to add acid then immediately add phenol. Therefore, we bypass exposing phenol to the initial heat. This protocol gave a better spectrum for blanks (Figure 2D). In addition, in all our future experiments, none of the blanks (carbohydrate free solutions) from this protocol turned orange.

We did not test the order to add glycogen. Our data in Figure 2B suggested phenol was the most sensitive to heat. We believed optimizing phenol's exposure to heat would impact the background and reproducibility more than glycogen's exposure to heat. Our glycogen and blank spectrum was of high quality (Figure 2D).

### 3.2 Generation of a response surface model

Our optimization journey first began by generating a numeric model that could predict absorbance with a given set of conditions. We logically selected our independent variables to be volume of phenol and volume of concentrated sulfuric acid. In addition, Rasouli et al. assert that the total signal is dependent on the total amount of aqueous volume (sample, water, and phenol) (Rasouli et al., 2014). Therefore, we also selected water to be an independent variable.

**FIGURE 3:**
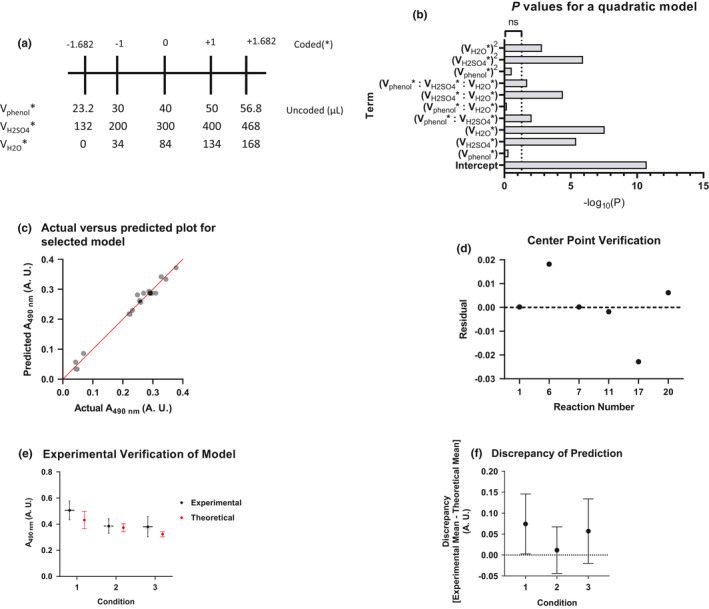
Optimization of a response surface model. (A) This diagram shows relevant coded values and their corresponding uncoded values. The specific experimental design is in Table 3. (B) The p‐values form Prism's hypothesis testing. Data were transformed with logarithms to facilitate visual comparison. (C) Hypothetical values were compared to experimental values to estimate goodness of fit for the selected model. (D) The residuals (data point minus mean) were calculated for each center point and plotted against the order the center point was run in the sequence. (E, F) Verification of response surface accuracy. For experimental data in black, all values are mean +/− 95% CI of the mean and is 4 replicates. For the model estimates, they are the estimated value +/− the 95% CI of the estimate. Panel F shows differences in the mean and the theoretical value +/− 95% CI

We first selected high and low volumes for each variable and encoded them so the response surface would be symmetric in all dimensions (Figure 3A). Our experimental design and the results of our data can be found in Table 3.

**TABLE 3:** Experimental setup and results

Run	A_490 nm_	V_phenol_*	V_H2SO4_*	V_H2O_*
1	0.292	0	0	0
2	0.344	−1	−1	−1
3	0.256	1	−1	−1
4	0.044	1	−1	1
5	0.043	0	−1.682	0
6	0.31	0	0	0
7	0.292	0	0	0
8	0.221	0	1.682	0
9	0.249	−1.682	0	0
10	0.378	0	0	−1.682
11	0.29	0	0	0
12	0.232	1	1	1
13	0.069	0	0	1.682
14	0.048	−1	−1	1
15	0.328	1	1	−1
16	0.26	−1	1	−1
17	0.269	0	0	0
18	0.224	−1	1	1
19	0.287	1.682	0	0
20	0.298	0	0	0

We initially fit the model to a full quadratic model and used GraphPad Prism's hypothesis testing to determine if specific terms were required (Figure 3B). We omitted the second order phenol term (V_phenol_*)^2^, and the interaction term between phenol and water (V_phenol_*: V_H2O_*). We left the primary phenol term (V_phenol_*) because some interactions, such as phenol and sulfuric acid (V_phenol_*: V_H2SO4_*), require a phenol term. We determined estimates for each parameter and their 95% CI (Table 4).

**TABLE 4:** Parameter estimates for our selected model

Parameter	Variable	Estimate	95% CI (asymptotic)
β0	Intercept	0.2869	0.2736 to 0.3002
β1	(V_phenol_*)	0.003508	−0.006887 to 0.01390
β2	(V_H2SO4_*)	0.04769	0.03730 to 0.05809
β3	(V_H2O_*)	−0.08491	−0.09531 to −0.07452
β4	(V_phenol_*: V_H2SO4_*)	0.021	0.007418 to 0.03458
β5	(V_H2SO4_*: V_H2O_*)	0.047	0.03342 to 0.06058
β6	(V_phenol_*: V_H2SO4_*: V_H2O_*)	−0.018	−0.03158 to −0.004418
β7	(V_H2SO4_*)^2^	−0.05295	−0.06302 to −0.04289
β8	(V_H2O_*)^2^	−0.02061	−0.03068 to −0.01054

We then used another approach to verify the correct terms were omitted. We had Prism compare the full quadratic model to the new model using an information theory approach – Akaike information criterion (AIC). When comparing our selected model to the full quadratic model, Prism calculated the difference in AICc to be −20.93 and a probability of >99.99% that our selected model is the correct when compared to a full quadratic model.

We plotted predicted A_490 nm_ for our selected model against our experimental data to observe the correlation (Figure 3C). The data agree well with the model, with *R* ^2^ being 0.9834. Large numbers of parameters in a model tend to describe data better just by random chance, thus require adjustments to *R* ^2^ to account for the number of variables. Our adjusted *R* ^2^ is 0.9713.

The experiment contained a built‐in quality control mechanism: the center points. Center points were spread throughout the experiment to monitor for potential process instability, such as decay of colored product during the experiment. The residuals were calculated and plotted against the order the reaction was run (Figure 3D). We observed no patterns in the residuals. We also calculated the mean absolute deviation around the mean to equal 0.008. We compared this to the mean of the center points (0.291) and found this ratio to be less than an acceptable 5%.

### 3.3 Experimental verification of the model's accuracy

Real response surfaces likely contain terms greater than a second degree; however, our model contained only second order terms since determining higher order effects would require many more data points and be harder to interpret. As a result, our second order model is likely to be an estimate of the real value. Consequently, we input some experimental conditions to test the predictive power of our model.

Our first condition was selected by using Excel's solver add‐in to find maximum signal. The results were near the extremes of all the variables which means this point was far away from experimental data. This will likely cause deviations from the model's prediction. However, the condition was still tested to see how the extreme the deviation is. The second condition tested was expected to be highly reproducible. The condition was estimated by calculating where the derivates of each component summed to the smallest amount. The third condition was randomly selected.

The confidence interval of the differences was calculated using the experimental data points (Figure 3E,F). For condition 1, the experimental minus theoretical difference was calculated to be 0.074 (95% CI: 0.003 to 0.150) A.U. The precision of the measurement is large which means the difference could be trivial (0.003 A.U.) or important (0.150 A.U.).

For Condition 2, the difference between the mean and the prediction was found to be 0.012 (95% CI: −0.044 to 0.066) A.U. For condition 3 the difference is 0.057 (95% CI: −0.020 to 0.134) A.U. One limitation should be noted: the confidence intervals were calculated based on the mean of the mean of the prediction and did not consider the confidence interval of the prediction itself.

### 3.4 Selection of precise conditions and verification

The mathematical model was used to optimize the assay, using Excel's Solver add‐in. We placed several constraints on the function. First, the total volume needed to be greater than 300 µL to ensure adequate volume for transfer to the microplate. We also constrained the volume of water to be 0 µl. We selected this condition because we wanted a simple protocol with fewer pipetting steps. To make this possible, we had to constrain the encoded water to be −1.682. This constraint means any of Solver's solutions would be outside the experimental range, likely causing the prediction to deviate from experimental values. We were okay accepting these slight deviations since any experimental conditions would be validated experimentally by generating calibration curves. We ultimately settled on 50 µl of sample, 0 µl DI water, 300 µl of concentrated sulfuric acid, and 45 µl of phenol.

Our mathematical modeling was done at the traditional 490 nm. This wavelength is selected to minimize interference from pentoses. We would be purifying out monosaccharides later, so this wasn't a concern. Before we analyzed all the curves, we took a representative spectrum of one of the glucose curves and used it to determine the wavelength that gave maximum signal (Figure S1).

**FIGURE 4:**
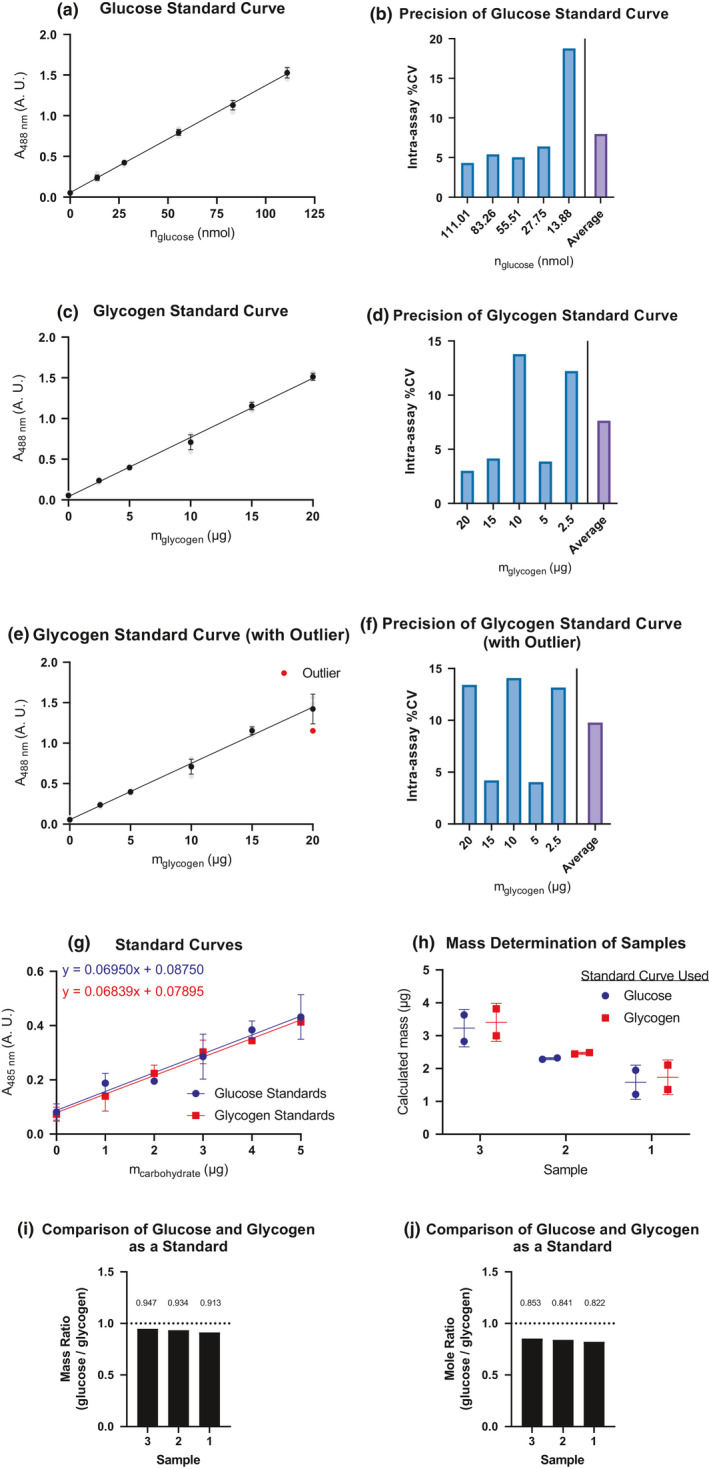
Standard curves and assay precision. (A) A standard curve was generated using glucose. (B) The precision of the glucose curve is represented as the %CV (standard deviation over the signal). (C) Shows a standard curve generated using glycogen. (D) This panel shows the precision of the glycogen curve. (E) The glycogen standard curve with the omitted outlier. (F) The %CV with the outlier and blanked with the new curve. (G–J) Show mass differences using glycogen and glucose as standards. (G) Sample standard curves generated using an un‐optimized protocol (50 μl sample, 300 μl H_2_SO_4_, 47 μl phenol followed by a 30‐min incubation before reading at 485 nm). All points were run in duplicate. (H) Samples of different masses were assayed. Their signal was analyzed using both the glycogen and glucose standard curve. The horizontal line is the average mass +/− the standard deviation. This panel illustrates how different curves lead to different outputs given the same input. (I). This figure shows the ratio of the average mass. We assume the ‘expected yield’ is the value from the glycogen curve and the ‘actual yield’ is the value from the glucose curve. Taking the mass ratio gives fractional yield. The number above each bar is the fractional value. Our data suggest a 91–95% yield based on mass, in agreement with observations by others (Passonneau & Lauderdale, 1974). (J) We repeat the comparison from (I), but use moles. We calculated the ‘actual yield’ from the mass of the glucose standard using the molar mass of glucose (180 g/mol). The molecular formula of glycogen is C_(6N)_H_(10N+2)_O_(5N+1)_ where N is the number of glucose molecules. This formula tells us that the molar mass of glycogen (162N + 18) g/mol where N is the number of glucose molecules stored in the glycogen particle. By assuming the molar mass of glycogen is ≈10^6^ g/mol (17), we can estimate there are ≈6172 glucose molecules per glycogen particle. From there, we calculate the expected number of moles of glucose (‘expected yield’) released from the glycogen assuming complete hydrolysis. (A, C, E, G, and H) show the mean +/− standard deviation. (A), (C), and (E) consists of 4 replicates except for the 20 μg condition in (C) which was done in triplicate due to the removed outlier. (G) and (H) were run in duplicate

The data were linear over a wide range and the intra‐assay %CV for each curve is under 10%. In sum, the %CV is around 5% for many of the individual points and the weighted averages are under 10% for each curve (Figure 4A–D). One outlier was removed from the glycogen data set due to experimental errors (Figure 4E,F). The intra‐assay %CV is under the acceptable 10% even when including this outlier.

We purposely express the units on the x‐axis differently with glycogen and glucose. When we compared a standard curve of glycogen to glucose and utilized mass as the x‐axis, we reproducibly saw glucose had larger slopes (Figure 4G,H). Such a result is not surprising since glucose and glycogen are different carbohydrate substrates for the reaction. We believe differences are likely to arise from two different sources:

1. Glycogen is likely not completely hydrolyzed into glucose (Figure 4I,J).
2. Mass differences between free glucose and glycosyl units in glycogen. Free glucose has a molar of mass of about 180 g/mol whereas a sugar unit in glycogen is about 162 g/mol due to loss of water during glycogen formation.

Because of these observations and the stoichiometric reasoning, we recommend that, when possible, a glycogen curve is used to quantify glycogen and a glucose curve is used to quantify glucose. A glucose standard can be used to quantify glycogen, but we recommend the units be reported as moles to eliminate mass from the interpretation and the reading be interpreted as “glucose equivalents” released, keeping in mind that not all the glycogen is hydrolyzed.

### 3.5 Selecting optimized precipitation steps

**FIGURE 5:**
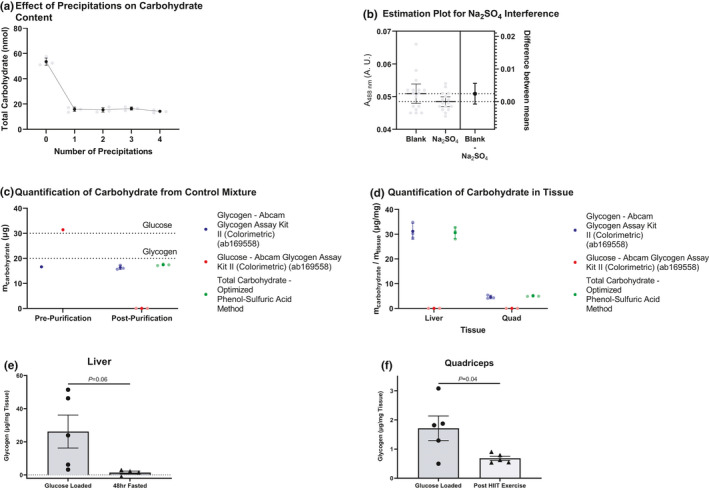
Determination and validation of the extraction conditions and of the method. (A) Shows the total carbohydrate in each sample after undergoing the noted number of precipitations. (B) The effect of sodium sulfate addition of signal was determined, and the difference was quantified. (C) Shows the total amounts of glycogen and glucose after undergoing one precipitation step and assayed using a commercial kit. Glucose masses were determined from the background glucose control. Zero masses were defined as negative mass after following the manufacturer's directions. Total carbohydrate of the final sample was also determined using the optimized phenol‐sulfuric acid procedure. Dashed lines represent theoretical values. (D) The sample procedure from part C was repeated but tissues from fasted rats infused with [U‐^13^C_6_] glucose were utilized instead of a simple, artificial carbohydrate mixture. (E) Liver glycogen content in mice loaded with an oral glucose bolus prior to study, as well as 48 h fasted mice. (F) Quadriceps muscle glycogen content in glucose‐loaded mice and their *ad lib* fed counterparts subjected to 1 h of high‐intensity interval training on the treadmill. (A, C, and D) show the mean +/− standard deviation. (A) is made from 4 replicates. (D) and (C) contain 3 replicates with the reception of the pre‐purification in (C) which contains a single measurement. (B) contains 95% confidence intervals. In panels (E) and (F), the 2‐tailed unpaired Student's *t*‐test was used to compare groups.

Glucose and glycogen are different and mass and are very insoluble in ethanol. We used ethanol precipitation and centrifugation to isolate glycogen from background carbohydrates, mainly glucose. We observed most of the carbohydrate content was removed after one precipitation/centrifugation cycle (Figure 5A). We therefore selected one to be the optimal number of steps.

### 3.6 Sodium sulfate interference

The purification and removal of background glucose signal introduced additional chemicals (Na_2_SO_4_) into the sample that were not present in the glucose/glycogen standards. We sought to determine whether this would affect the signal of the assay.

We ran our optimized assay protocol without glucose. Instead, we used 50 µl of the concentrated sodium sulfate solution. This situation represents a ‘worst‐case’ scenario. In the assay procedure, glycogen is precipitated with 50 µl of the saturated sodium sulfate solution. After the end of the purification, any sodium sulfate recovered is redissolved into a few hundred microliters of water, diluting the sodium sulfate. Therefore, even if all sodium sulfate is recovered, the final solution is much more dilute than the stock we used to run these controls.

To analyze our data, we calculated the difference of the means and the confidence interval for this difference. We could not detect a difference between the two samples at the 95% confidence (Figure 5B).

### 3.7 Checking for glucose removal and glycogen retention

Our experiment to optimize precipitation steps did not tell us how much glucose remained in each sample or how much glycogen as removed. We therefore used a commercial enzymatic essay specific for glycogen. We tested a sample of glucose/glycogen mixture that underwent the purification procedure and sample that did not undergo the extraction procedure. This control allowed us to assess extraction efficiency. We also compared the final glycogen content to the optimized phenol‐sulfuric acid method. Our results show that one step removes all the glucose, does not remove glycogen, and the phenol‐sulfuric acid method output is the recovered glycogen (Figure 5C).

### 3.8 Validation with mouse tissue

Tissues are more complicated mixtures than the simple and artificial glucose/glycogen mixture. So, we tested the extraction procedure on tissues. We utilized the same enzymatic assay to test for glucose and glycogen. We also ran the samples with the optimized phenol‐sulfuric acid protocol. Like the simple glucose/glycogen mixture, we noticed a complete removal of glucose and excellent agreement between the phenol‐sulfuric acid method and the enzymatic assay (Figure 5D). We concluded that one extraction step removes all the simple carbohydrates, leaving behind solely glycogen that can be read out by the phenol‐sulfuric acid method.

### 3.9 Application of the method under physiologically variant conditions

Finally, we aimed to confirm the ability of the method to detect differences in glycogen content under conditions expected to generate a wide variation in liver and muscle glycogen content: fed and fasted conditions, as well as an intense bout of treadmill exercise. As expected, liver glycogen strongly tended (*p *= 0.06) to decrease in 48 h fasted as compared to glucose loaded mice, and quadriceps glycogen concentrations were significantly reduced in mice following exercise as compared to their glucose loaded, sedentary counterparts (Figure 5E,F).

## 4 DISCUSSION

There has been substantial debate regarding the optimal method to measure tissue glycogen (Passonneau & Lauderdale, 1974). In this study, we sought to optimize a protocol that would allow reproducible measurement of tissue glycogen including under conditions of low glycogen content. Several experimental conditions were varied in the current study. The first question was whether or not a heating step is of utility, as some (Masuko et al., 2005; Michel et al., 1956; Robyt, 2008) but not all (Rao & Pattabiraman, 1989; Rasouli et al., 2014) reported protocols involve a heating step. Our results suggest that adding even a brief heating step can cause the phenol to give inconsistent background signal. Using the heat released by addition of acid gave more consistent results. In a similar manner, Taylor found that manipulating temperatures gives variable results on the conversion of monosaccharides to furfurals and thus colored products (Taylor, 1995). Therefore, we selected to use the heat of dilution to drive the reaction.

We also tested the order to add the reagents. We tested two options: the protocol from Rasouli et al. (add phenol then acid) (Rasouli et al., 2014) and the protocol from Masuko et al. (add acid then immediately add phenol) (Masuko et al., 2005). Both methods gave low background signal but the Masuko et al. protocol gave more reproducible values. Addition of acid causes a violent release of heat, occasionally causing the solution to briefly boil or for vapor to be released. We postulate the violent heat release could cause the phenol, if present, to form small quantities of unwanted products or to boil out of the solution. Therefore, we bypass exposing phenol to the initial heat. We selected to add acid then immediately add phenol. In all our future experiments, none of the blanks (carbohydrate free solutions) from this protocol turned orange.

We next proceeded to optimize the phenol‐sulfuric acid protocol. We selected a specific set of reactions – following a central composite design – that optimized the predictive power of our model. A test set of data never seen by our model predicted the values well for points near the curve. We were able to use this to generate a model that has good precision (%CV ranging from 5 to 10%) and highly sensitivity. Our glycogen protocol has about twice the sensitivity of a similar glycogen protocol developed by Rasouli et al. (2014).

Background glucose is a challenge for any measurement of glycogen. Enzymatic methods typically require measuring two samples. One sample contains the background glucose and hydrolyzed glycogen while the other sample contains the intact glycogen and background glucose. The glycogen content is found by subtracting the total glucose from the background glucose. This method is attractive because it allows estimation of background glucose but this costs precision of the glycogen measurement. According to error propagation, the error in the glycogen measurement will depend on the error in the two samples measured. In comparison, our method uses centrifugation to physically separate the glycogen from the background glucose. We sacrifice the ability to measure the free glucose, but the uncertainty in our glycogen measurement is not the sum of two measurements. We also believe that the glucose‐free glycogen solution can be a starting point for further experiments, such as mass spectrometry or analysis of molecular size distributions.

Our method generates a glucose‐free glycogen pellet that gives the experimenter flexibility. Our results show the added Na_2_SO_4_ is not a concern for the assay, as even in the ‘worst‐case’ scenario where all Na_2_SO_4_ is recovered, there is no interference. Therefore, the glycogen pellet can be dissolved into small volumes of a few hundred microliters, creating a concentrated sample that can be detected with our sensitive phenol‐sulfuric acid protocol.

We tested our extraction procedure against a commercial enzymatic assay that utilizes the amyloglucosidase method. Our method gives results in agreement with the commercial kit and with comparable standard deviations. Assay kits are convenient because all materials arrive together with a protocol that has already done various calculations for the user. These assay kits, however, can easily range in cost from $4–8 per sample and often include a small number of tests (100 to 500). Our assay enables the user to purchase materials separately and perform calculations on their own. Although more work upfront, this provides a long‐term cost and time savings. For example, if a user makes a solution using 25 g of phenol crystals and aliquots the preparation, they can make enough solution for over 10,000 tests.

With our highly sensitive phenol‐sulfuric acid assay and our optimized extraction protocol, we decided to test the ability of our setup to detect glycogen under a variety of physiological conditions. Mouse models were used since mice offer less tissue than humans or rats. Therefore, a highly sensitive assay is needed to detect glycogen changes. Using samples from mice under conditions expected to vary glycogen content (glucose loading, a prolonged fast, and an intense bout of treadmill exercise), we verified the efficacy of the method to detect differences even in the low range of glycogen concentrations typically observed in muscle.

In sum, we have successfully applied a multi‐variable optimization approach to develop a precise and highly sensitive, inexpensive phenol‐sulfuric acid protocol which we coupled to a simple and flexible glycogen extraction protocol. We believe our method will be useful for detecting glycogen in tissues or cells, even under conditions of glycogen depletion.

## CONFLICT OF INTEREST

None of the authors has any conflict of interest relevant to this study.

## AUTHOR CONTRIBUTIONS

The study was conceived by K.J.S. Experiments were performed by K.J.S. and B.P.L. The manuscript was written by K.J.S. and edited by B.P.L. and R.J.P.

## Supporting information



Figure S1
