# Supplementary figures and images for: An optimized method for tissue glycogen quantification
Source: Physiol Rep. 2022 Feb 18;10(4):e15195. doi: 10.14814/phy2.15195 (PMC8855679; doi:10.14814/phy2.15195)

# Representative Glucose Spectrum

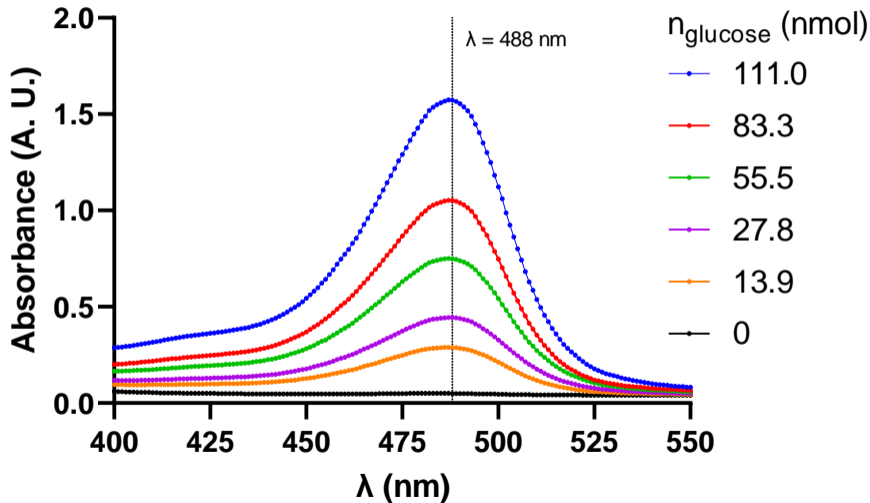

Supplement: Supplementary file 1 — Figure S1 [file PHY2-10-e15195-s001.pdf]
